# A Case of Legionnaires’ Disease Manifesting as Heat Exhaustion

**DOI:** 10.7759/cureus.35099

**Published:** 2023-02-17

**Authors:** Muhammad Ahmed Malik, Fahd Shaukat, Aqsa Malik, Syeda Kaifee, Mohamed Eid

**Affiliations:** 1 Medicine, Rochester Regional Health, Rochester, USA; 2 Medicine, Fatima Memorial Hospital College of Medicine and Dentistry, Lahore, PAK; 3 Medicine, Lake Erie College of Osteopathic Medicine, Erie, USA

**Keywords:** heat exhaustion, pneumonia, pontiac fever, legionnaires’ disease, legionellosis

## Abstract

A 69-year-old male with a history of prior admissions of heat exhaustion presented with non-specific symptoms including fatigue, diarrhea, and dehydration. The workup revealed a positive legionella urine antigen. He was treated with levofloxacin with symptom resolution within 48 hours.

## Introduction

Legionnaires’ disease ranges from mild to severe pneumonia. Legionella species are usually acquired from contaminated water sources and soil. The outbreaks usually occur in the summer and fall seasons. Common clinical features that raise suspicion for Legionnaires’ disease are respiratory and gastrointestinal manifestations, hyponatremia, and transaminitis. We present a case of Legionnaires’ disease manifesting in a patient with heat exhaustion.

## Case presentation

**Figure 1:**
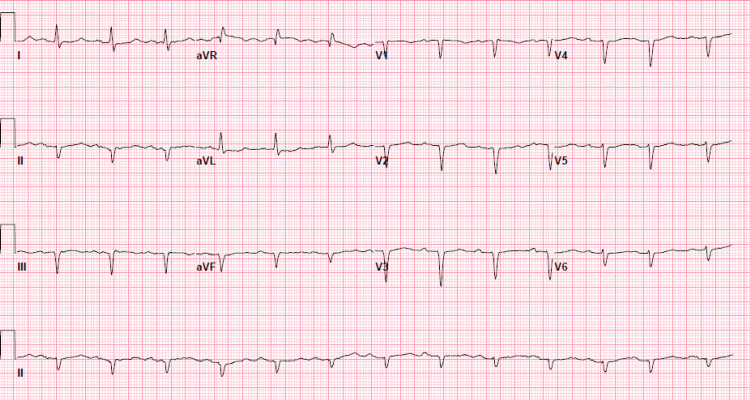
EKG showing normal sinus rhythm with first-degree AV block. EKG: Electrocardiogram; AV: atrioventricular

**Figure 2:**
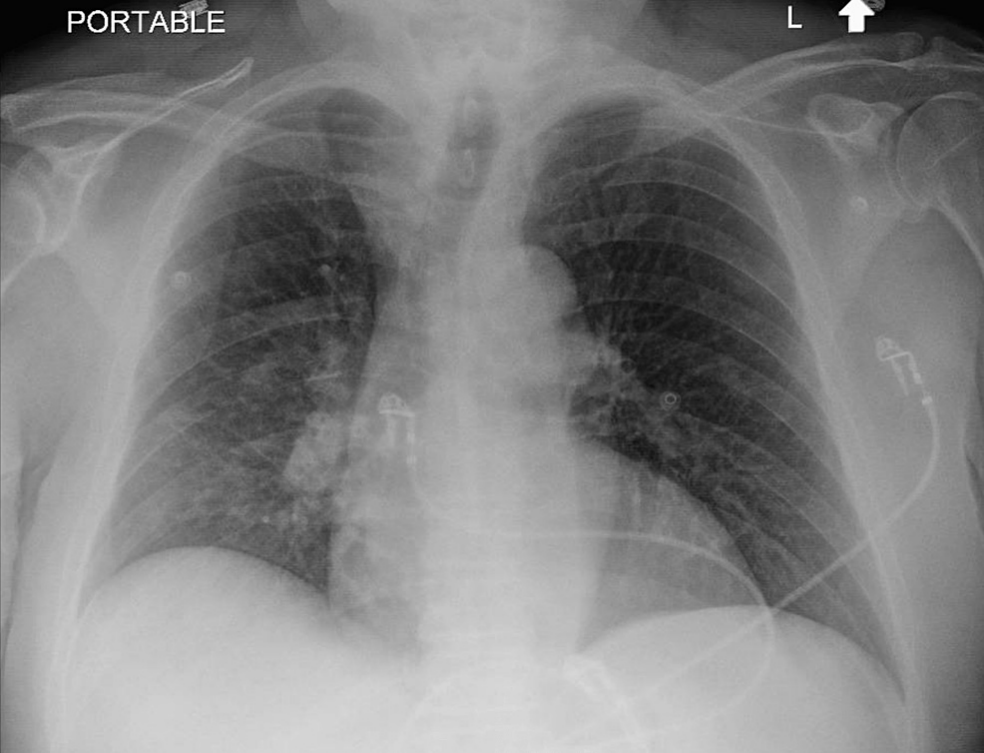
Chest X-ray shows evolving right lower lobe opacity.

A 69-year-old male with a history of bipolar disorder and hypertension presented to the emergency department in July with a one-week history of malaise, fatigue, diarrhea, and dehydration. He was a farmer by profession and usually experienced these symptoms while working outside during the summertime each year. This time, he also attributed his symptoms to dehydration and heat exhaustion. On presentation to the emergency department, his blood pressure was 95/60 mmHg and his pulse was 100 beats per minute. He was afebrile with a T-max of 98.2 F. Physical examination was consistent with an ill-appearing gentleman having dry mucous membranes and dry skin with poor turgor. Admission labs were significant for leukocytosis (WBC 12.1 x 10^3^ /ul; ref range 4.0-10.8 x 10^3^/ul), hyponatremia (Na 127 mEq/L; ref range 135-145 mEq/L), hypokalemia (K 2.7 mEq/L; ref range 3.5-5.1 mEq/L), hypochloremia (Cl 91 mEq/L; ref range 98-107 mEq/L), hypomagnesemia (Mg 1.7 mg/dL; ref range 1.6-2.6 mg/dl), elevated creatinine 1.80 mg/dl (baseline creatinine around 1.40 mg/dl), and mild transaminitis (AST 116 U/L; ref range 7-37 U/L; ALT 47 U/L; ref range 10-47 U/L). His lipase was 15 U/L (ref range 12-53 U/L). Ultrasound abdomen was unremarkable. The electrocardiogram (EKG) showed normal sinus rhythm with first-degree atrioventricular block (Figure 1). High-sensitivity troponins were unremarkable. Given hyponatremia, serum osmolality and urine studies were conducted, and their results were consistent with hypotonic hypovolemic hyponatremia. Based on prior history of heat exhaustion, examination, and laboratory findings, he was admitted as a case of severe dehydration secondary to heat exhaustion and started on IV hydration and electrolyte repletion. The patient initially felt better with these interventions. However, within six hours of admission, he developed a fever with a T-max of 102.3 F with chills. Physical examination was notable for mild respiratory distress, but his oxygen saturation was above 95% on room air. Chest X-ray was obtained which showed evolving opacity in the right lower lobe (Figure 2). The patient was now meeting the systemic inflammatory response syndrome criteria; he was started on empiric ceftriaxone and azithromycin for presumed community-acquired pneumonia. Due to a combination of possible pneumonia, hyponatremia, and transaminitis on admission labs, legionella urine antigen was ordered and surprisingly came back positive. He was diagnosed with Legionnaire’s disease. The antibiotic therapy was readjusted to levofloxacin. On detailed history taking, no source of contamination was identified, and it was concluded that the patient might have acquired the infection while working outside on farms. Within 48 hours, the patient’s diarrhea, transaminitis, and respiratory systems subsided. He was discharged on a 10-day course of levofloxacin. He remained well post-discharge.

## Discussion

Legionella infections commonly occur as outbreaks in the summer and fall seasons [1,2]. The symptoms of Legionnaires’ disease are very non-specific ranging from pneumonia to severe gastrointestinal and electrolyte abnormalities [3]. It should also be considered in patients with pneumonia who are not responding to standard beta-lactam monotherapy [4]. Legionella infection can manifest in two different forms collectively known as Legionellosis [5].

Legionnaires’ disease

The symptoms usually begin within 2-14 days after exposure to bacteria. This disease appears very similar to usual community-acquired pneumonia. The usual symptoms are fever, cough, dyspnea, muscle aches, and headaches.

Pontiac fever

This is a mild form of the disease and usually presents with fever and muscle aches only. It is not associated with pneumonia. The symptoms begin within few hours to three days after exposure to contaminated water sources.

A previous multi-center study with patients from four different hospitals introduced a six-point predictive score for Legionella to aid in diagnosis. This criterion has a good specificity of around 93.1% and assesses temperature, dry cough, hyponatremia, LDH, CRP, and platelets in affected patients. This proposed score can be considered as one method to efficiently diagnose and initiate targeted antibiotic treatment in patients with suspected infection [6]. Early diagnosis with polymerase PCR or urine legionella antigen is very important in suspected cases. Respiratory cultures usually take time to grow and delay the diagnosis. Early treatment is crucial to improve the outcomes and avoid mortality in such cases [7]. Macrolides and respiratory fluoroquinolones are the first-line treatment options. Patients with Legionnaires’ disease usually respond to therapy within 48 hours and usually require a 7-10-day course [8]. Pontiac fever usually does not require antimicrobial therapy and can be managed conservatively with close monitoring.

## Conclusions

Legionellosis commonly occurs as an outbreak in the summer and fall seasons. The disease manifests from simple pneumonia to multiorgan involvement. High suspicion, timely diagnosis, and early treatment have shown improved outcomes including earlier symptom resolution and decreased length of hospital stay and mortality. 

Per the literature review, usually, the disease is acquired from contaminated water sources and typically presents with respiratory and gastrointestinal manifestations. Our case is unique since the patient presented with very nonspecific symptoms resembling heat exhaustion, but a timely diagnosis of legionnaires’ disease and treatment led to symptom resolution within 48 hours.
